# The Associations of rs1799724 and rs361525 With the Risk of Ankylosing Spondylitis Are Dependent on HLA-B27 Status in a Chinese Han Population

**DOI:** 10.3389/fimmu.2022.852326

**Published:** 2022-04-05

**Authors:** Nan Sheng, Yingying Gao, Hui Li, Wenwen Wang, Linyu Geng, Bo Zhang, Qiang Huang, Xueqin Wang, Lingyun Sun

**Affiliations:** ^1^ Department of Rheumatology, Affiliated Hospital 2 of Nantong University and First People’s Hospital of Nantong City, Nantong, China; ^2^ Clinical Medicine Research Center, Affiliated Hospital 2 of Nantong University and First People’s Hospital of Nantong City, Nantong, China; ^3^ Department of Rheumatology, Drum Tower Clinical Medical College of Nanjing Medical University, Nanjing, China; ^4^ State Key Laboratory of Environmental Geochemistry Institute of Geochemistry, Chinese Academy of Science, Guiyang, China

**Keywords:** ankylosing spondylitis, tumor necrosis factor alpha, single nucleotide polymorphism, human leucocyte antigen B27, early diagnosis, risk prediction

## Abstract

**Objectives:**

Human leucocyte antigen B27 (HLA-B27) is an important biomarker for ankylosing spondylitis (AS). However, delay in the diagnosis of AS is still common in clinical practice. Several single nucleotide polymorphisms (SNPs) in the coding gene of tumor necrosis factor alpha (TNFα) have been reported to be AS susceptibility loci. Our aim was to explore whether SNPs in *TNFα* could be used to improve the performance of HLA-B27 for predicting AS.

**Methods:**

Five SNPs (rs1799964, rs1800630, rs1799724, rs1800629, and rs361525) spanning *TNFα* were genotyped by qPCR-Invader assay in 93 AS patients and 107 healthy controls for association analysis and linkage disequilibrium (LD) analysis. Random forest algorithm was utilized to construct the predictive classifiers for AS. *HLA-B* was genotyped by PCR-sequence-based typing in a subset of the HLA-B27-positive subjects (38 AS patients and 5 healthy controls).

**Results:**

The T allele of rs1799724 was verified to significantly increase the risk of AS (OR = 4.583, *p* < 0.0001), while the A allele of rs361525 showed an association with the reduced AS risk (OR = 0.168, *p* = 0.009). In addition, the rs1799964^T^-rs1800630^C^-rs1799724^T^-rs1800629^G^-rs361525^G^ haplotype was significantly associated with a higher risk of AS (*p* < 0.0001). The optimal set of variables for classifiers to predict AS only consisted of HLA-B27. Strong associations with HLA-B27 status were found in both rs1799724 (*p* < 0.0001) and rs361525 (*p* = 0.001), and all the analyzed HLA-B27-positive subjects carried HLA-B*27:04 or HLA-B*27:05.

**Conclusion:**

In the Chinese Han population, the minor allele T of rs1799724 could increase the risk of AS, while the minor allele A of rs361525 protects individuals from AS. However, the contributions of rs1799724 and rs361525 to AS risk were dependent on HLA-B27 status, suggesting the importance of taking the independence and specificity into consideration in AS susceptibility loci studies.

## Introduction

Ankylosing spondylitis (AS), a radiographic form of axial spondyloarthritis, is a chronic inflammatory autoimmune disease that affects new bone formation (1, 2). The main clinical feature of AS is chronic back pain, which could result in serious impairments of spinal structure and physical function with a negative impact on the quality of life (3). According to the estimated global prevalence, the average prevalence of AS per 100,000 was 0.74 to 3.19 (4). The treatment of AS has been greatly improved in the decades; however, delayed diagnosis remains a formidable obstacle (5, 6). Due to the limitations of the primary diagnosis of AS in daily practice, the duration of delay ranges from 5 to 11 years in different regions (6, 7). Therefore, timely and specific diagnosis of AS is crucial for good treatment outcome, preventing the worse functional impairment in the spine.

Currently, the commonly used measurement for diagnosing AS includes diagnostic imaging, which have inherent limitations for early prediction (8). Previous studies showed that AS was a heritable disease with heritability in twins of over 90% (9, 10); hence, genetic factors could be potential biomarkers for screening of AS. Although human leukocyte antigen B27 (HLA-B27), or the coding gene HLA-B*27, emerges as a diagnostic biomarker for AS, it only contributes ~20% of the genetic susceptibility to AS (3, 11). An urgent need exists for better biomarkers or prediction methods for early screening and management of high-risk population with AS.

In addition to HLA-B27, single nucleotide polymorphism (SNP) loci have been discovered as susceptibility factors of AS benefiting from the rapid development of genetic studies, especially genome-wide association study (GWAS) (12, 13). However, a majority of alleles of the reported susceptibility loci have a modest effect on the risk of AS and are poorly reproducible in other groups. Minor allele frequencies of SNP, usually the risk allele, could be various from different ancestries (14); e.g., *PTPN22* risk allele (1858C>T) was found and verified the association with AS in Europe, while the risk allele frequency was much lower and nearly absent in Asia (15, 16). Given the inconsistency of the discovered susceptibility loci and the influence of population ancestry, observational studies taking ethnic origins into account are needed for further confirmation. Moreover, multiple susceptibility factors should be integrated to improve the predictive power of AS. Genetic risk score (GRS) is a measure to calculate the cumulative effect of multiple SNPs and has been used for susceptibility in rheumatic disease including AS (17). However, GRS relies on a large number of SNPs, which are usually selected from GWAS that require further validation. Therefore, both the accuracy and the cost of GRS are unsatisfactory. An ideal analysis tool for AS susceptibility should be specific and accurate with the easily available disease-specific markers.

Tumor necrosis factor alpha (TNFα) is a vital proinflammatory cytokine of the fragile communication networks of cytokines in various chronic inflammatory diseases (18). TNFα mRNA was first detected in sacroiliac joints in 1995, suggesting the association between TNFα and the pathogenesis of AS (19). Some SNPs, reported as susceptibility loci, in *TNFα* and the coding gene of its receptors (*TNFRSF1A* and *TNFRSF1B*) could alter gene transcription or protein function, which could partially account for the pathogenesis of AS in genetic factors (20, 21). As most of the reported susceptibility loci, the association between the SNPs and the risk of AS are controversial in different geographic regions and ethnicities (22). Here, we focused on five SNPs in *TNFα* (rs1799964, rs1800630, rs1799724, rs1800629, and rs361525) for further verification in a Chinese Han population, and investigated whether they contributed to AS independently that could be a good complement for HLA-B27 in AS prediction.

## Materials and Methods

### Study Subjects

The association analysis of SNPs and disease was conducted on 93 AS patients and 107 healthy controls. Additional group consisting of 18 AS patients, 20 patients with lumbar disc herniation (LDH), and 20 healthy controls were recruited for validation. All of the subjects were Chinese Han population and unrelated to each other. AS patients were attending the outpatient rheumatology department of the First People's Hospital of Nantong City. All of the AS patients (1) met the Assessment in SpondyloArthritis international Society (ASAS) classification criteria for axial spondyloarthritis (23); (2) had radiographic sacroiliitis; and (3) were being treated with TNFα blockade after no obvious remission by using non-steroidal anti-inflammatory drugs (NSAIDs) for more than 4 weeks. Patients with LDH and controls were recruited from the volunteers who have passed the physical examination, excluding autoimmune and/or rheumatic diseases. All the participants gave written informed consent to the study, which was approved by the Ethics Committee of First People’s Hospital of Nantong City and performed according to the principles of the Declaration of Helsinki.

### Demographic and Clinical Data Collection

Characteristics including gender, age, age of symptom onset, body mass index (BMI), family history and disease duration, bath ankylosing spondylitis disease activity index (BASDAI), and bath ankylosing spondylitis functional index (BASFI) were obtained from self-administered questionnaires by patients or controls. Clinical features including HLA-B27, serum levels of erythrocyte sedimentation rate (ESR), and high-sensitivity C-reactive protein (hs-CRP) were collected from medical records. HLA-B27 detection of AS patients was performed by flow cytometry and that of healthy controls and LDH patients was fulfilled by TaqMan assay (Jiangsu Weihe Biotechnology INC, China).

### Genotyping

Genomic DNA from patients and controls was prepared as samples from EDTA-stabilized peripheral blood by Wizard^®^ Genomic DNA Purification Kit (Promega Corporation, USA) according to the manufacturer’s protocols. The genomic DNA was quantified by NanoDrop (Thermo Fisher Scientific, USA) with the concentration over 40 ng/μl and A260/280 between 1.8 and 2.0.

All the samples were genotyped for five SNPs in *TNFα* (rs1799964, rs1800630, rs1799724, rs1800629, and rs361525) by using the previous reported qPCR-Invader (24). Briefly, a final volume of 20 μl of reaction mixture for each SNP genotyping contained 1× reaction buffer (pH 8.0, 7.5 mM of MgCl_2_), 0.25 mM of dNTP mixture, 0.5 μM of each primer, 1 U of *Taq* DNA polymerase, 400 U of *Afu* flap endonuclease, 50 nM of upstream probe, 60 nM to 250 nM of each allele-specific downstream probe, 250 nM of each fluorescence resonance energy transfer (FRET) probe, and 1 μl of the target (at least 1 ng of genomic DNA). The amplification conditions consisted of a pre-denaturation at 95°C for 3 min, followed by 10 cycles of 95°C for 15 s, 67°C for 30 s, and 70°C for 20 s, and followed by 35 cycles of 95°C for 15 s, 63°C for 1 min (detecting the fluorescence signal), and 70°C for 20 s by using 7500 Fast Real-Time PCR System (Thermo Fisher Scientific, USA). The sequences of each SNP were obtained from Database of Single Nucleotide Polymorphisms (https://www.ncbi.nlm.nih.gov/snp/), and the sequences of primers and detection probes are listed in **Supplementary Table S1**. Five percent of samples were further verified by PCR resequencing. *HLA-B* genotyping was performed by PCR-sequence-based typing (Jiangsu Weihe Biotechnology INC, China).

### Statistical Analysis

SPSS version 25.0 software and SAS version 9.4 were employed for statistical analysis. According to the results of normality distribution by Kolmogorov–Smirnov test, Chi-square test, *t*-test, or Mann–Whitney *U* test was used to compare the demographic and clinical data. Continuous data with normal distribution were displayed as mean ± standard deviation (SD); otherwise, data were presented as median (interquartile range, IQR). The genotype, allele, and genetic models of each SNP were compared in patients and controls by using Chi-square test or Fisher’s exact test where appropriate. Bonferroni correction was used for multiple comparisons. Odds ratios (ORs) with 95% confidence intervals (95% CIs) were calculated for the association assessment. Kruskal–Wallis test was used to compare independent groups. Hardy–Weinberg equilibrium (HWE), linkage disequilibrium (LD), and haplotype analysis were implemented using SHEsis software online (25). A two-sided *p-*value of less than 0.05 was considered statistically significant.

### Establishment of the Predictive Classifier for AS

The random forest algorithm (R version 3.6.1, R package of randomForest 4.6) was conducted for constructing a classifier for AS and analyzing the contribution of the predictive variables. The training group was consisted of 93 AS patients and 107 healthy controls used in the association analysis, while the validation group was formed by the new recruited subjects. Due to the similar symptoms and inappropriate overuse of MRI, patients with AS could be misdiagnosed as LDH especially in the early stage (26). We recruited patients with LDH as negative samples besides healthy subjects to investigate the specificity of classifier; therefore, the validation group consisted of 18 AS patients, 20 patients with LDH, and 20 healthy subjects. The set of variables for the classifier included gender, family history, the five SNPs, and HLA-B27. The classifier could output the probability of AS after inputting the variables. We used the area under the receiver-operating characteristic curve (AUC) to assess the performance of the classifier. Fivefold cross-validation was conducted for the errors with different variables. To evaluate the contribution of each variable to AS, mean decrease accuracy and mean decrease Gini were utilized in this study.

## Results

### Demographic and Clinical Characteristics

The delayed diagnosis of ~7 years was observed in the AS patients since the median age of symptom onset was 30.00 (IQR 17.00) years and the median age of diagnosis was 37.00 (IQR 18.50) years (**Table 1**). The positive rate of HLA-B27 in AS patients was 95.7%, confirming the strong association of HLA-B27 positivity with AS. Besides, it showed that both the median age of symptom onset [25.00 (IQR 7.50) years vs. 33.00 (IQR 19.00) years, *p* = 0.005] and the median age of diagnosis [28.00 (IQR 11.75) years vs. 38.00 (IQR 19.00) years, *p* = 0.024] were earlier in patients a with family history than without (**Supplementary Table S2**).

**Table 1:** Demographic and clinical characteristics of ankylosing spondylitis (AS) patients.

Characteristic	AS (*n* = 93)	HC (*n* = 107)	*p*-value
Gender (male, %)	69.9	57.0	0.060
Age at enrollment [years, median (IQR)]	37.00 (18.00)	31.00 (16.00)	0.095
Age at symptom onset [years, median (IQR)]	30.00 (17.00)	N/A	N/A
Age at diagnosis [years, median (IQR)]	37.00 (18.50)	N/A	N/A
BMI [kg/m^2^, mean ± (SD)]	23.88 ± 3.28	24.24 ± 3.49	0.450
Family history of AS (%)	15.1	0	**<0.0001**
HLA-B27 positivity (%)	95.7	4.7	**<0.0001**
ESR [mm/h, median (IQR)]	16.00 (41.00)	N/A	N/A
Hs-CRP [mg/L, median (IQR)]	13.12 (37.62)	N/A	N/A
BASDAI [score, median (IQR)]	3.30 (2.10)	N/A	N/A
BASFI [score, median (IQR)]	2.80 (1.95)	N/A	N/A

AS, AS patient; HC, healthy control; BMI, body mass index; ESR, erythrocyte sedimentation rate; Hs-CRP, high-sensitivity C-reactive protein; BASDAI, bath ankylosing spondylitis disease activity index; BASFI, bath ankylosing spondylitis functional index; N/A, not applicable. The bold value showed the significance level less than 0.05.

### Genotype Distributions, Allele Frequencies, and Inheritance Models

All of the five SNPs were tested for HWE in AS patients and healthy controls, and none of them deviated from HWE (**Supplementary Table S3**, *p* > 0.05). The distributions of genotypes and allele frequencies of all the SNPs are shown in **Table 2**. Among the SNPs, only rs1799724 (*p* < 0.0001) and rs361525 (*p* = 0.007) showed significant differences in genotype distributions between AS patients and healthy controls, even after Bonferroni correction. Moreover, the T allele of rs1799724 showed a higher risk of AS than C allele (OR = 4.583, 95% CI: 2.853–7.363, *p* < 0.0001, the corrected *p* < 0.0001). The A allele of rs361525 showed a lower risk of AS than the G allele (OR = 0.168, 95% CI: 0.037–0.755, *p* = 0.009, the corrected *p* = 0.045). According to the analysis of inheritance models, there were significant differences between AS patients and healthy controls in the over-dominant model, dominant model, recessive model, and co-dominant model (**Supplementary Table S4**, all *p* < 0.001), suggesting a strong association between rs1799724 and AS susceptibility.

**Table 2:** Genotypes and allele frequencies of *TNFα* polymorphisms.

SNP	Genotype or allele	AS, *n* (%)	HC, *n* (%)	*p*-value	Corrected *p*-value	OR (95% CI)
rs1799964	TT	71 (76.3)	71 (66.4)			Reference
	TC	18 (19.4)	31 (29.0)	0.268^a^	0.136^c^	0.595 (0.300–1.178)
	CC	4 (4.3)	5 (4.7)		0.719^c^	0.777 (0.197–3.065)
	T	160 (86.0)	173 (80.8)	0.166^b^	0.830^d^	Reference
	C	26 (14.0)	41 (19.2)			0.686 (0.401–1.172)
rs1800630	CC	72 (77.4)	81 (75.7)			Reference
	CA	19 (20.4)	23 (21.5)	0.954^a^	0.937^c^	0.972 (0.479–1.971)
	AA	2 (2.2)	3 (2.8)		0.617^c^	0.625 (0.099–3.939)
	C	163 (87.6)	185 (86.4)	0.725^b^	1^d^	Reference
	A	23 (12.4)	29 (13.6)			0.900 (0.501–1.618)
rs1799724	CC	25 (26.9)	76 (71.0)			Reference
	CT	53 (57.0)	30 (28.0)	**<0.0001** ^b^	**<0.0001** ^c^	4.953 (2.596–9.447)
	TT	15 (16.1)	1 (1.0)		**<0.0001** ^c^	46.377 (5.767–372.962)
	C	103 (55.4)	182 (85.0)	**<0.0001** ^b^	**<0.0001** ^d^	Reference
	T	83 (44.6)	32 (15.0)			4.583 (2.853–7.363)
rs1800629	GG	86 (92.5)	94 (87.9)	0.277^b^	0.297^c^	Reference
	GA	7 (7.5)	13 (12.1)			0.592 (0.221–1.587)
	G	179 (96.2)	201 (93.9)	0.290^b^	1^d^	Reference
	A	7 (3.8)	13 (6.1)			0.605 (0.236–1.549)
rs361525	GG	91 (97.8)	94 (87.9)	**0.007** ^b^	**0.021** ^c^	Reference
	GA	2 (2.2)	13 (12.1)			0.165 (0.035–0.760)
	G	184 (98.9)	201 (93.9)	**0.009** ^b^	**0.045** ^d^	Reference
	A	2 (1.1)	13 (6.1)			0.168 (0.037–0.755)

a Fisher’s exact test.

b Chi-square test.

c adjusted by sex and age.

d corrected for multiple comparisons by Bonferroni correction. HC, healthy control; OR, odds ratio; CI, confidence interval. The bold value showed the significance level less than 0.05.

### Linkage Disequilibrium and Haplotype Analysis

**Figure 1:**
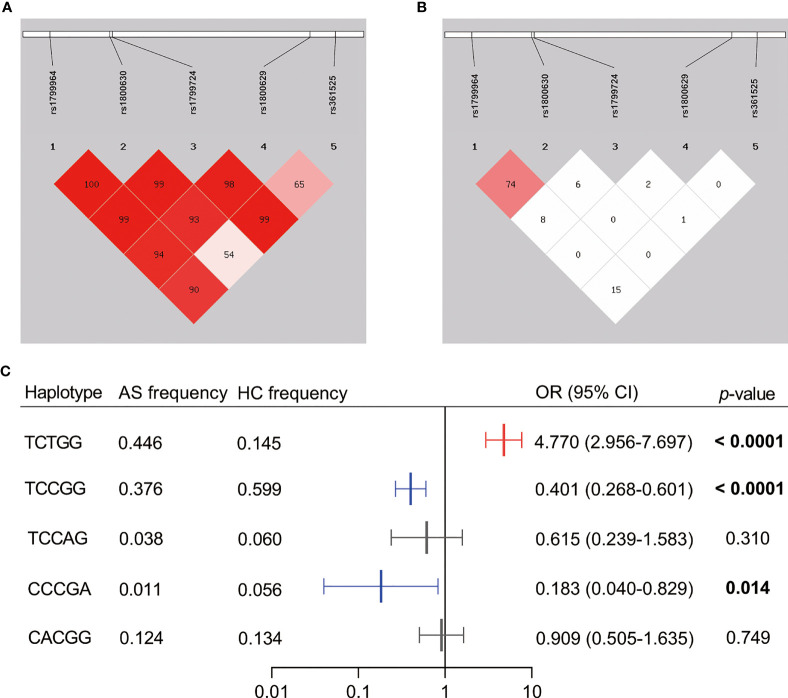
Linkage disequilibrium (LD) pattern and haplotype analysis of the five SNPs genotyped in *TNFα*. **(A)** The plot shows *D*’ values in percentage as a pairwise measure of LD. **(B)** The plot shows *r* ^2^ values in percentage as a pairwise measure of LD. **(C)** Haplotypes were constructed by rs1799964, rs1800630, rs1799724, rs1800629, and rs361525. Dark shading indicates strong LD. The haplotype analysis was omitted if the frequencies were less than 0.03. AS, ankylosing spondylitis patients; HC, healthy control; OR, odds ratio; CI, confidence interval. The bold value showed the significance level less than 0.05.

After the linkage disequilibrium (LD) analysis of the five SNPs in *TNFα* (**Figures 1A, B**), we observed a strong LD between rs1799964 and rs1800630 both in the assessment by *D*’ value (*D*’ = 1) and *r* ^2^ value (*r* ^2^ = 0.74). As shown in **Figure 1C**, a further haplotype analysis demonstrated that the total frequency of the rs1799964^T^-rs1800630^C^-rs1799724^C^-rs1800629^G^-rs361525^G^ haplotype and the rs1799964^T^-rs1800630^C^-rs1799724^T^-rs1800629^G^-rs361525^G^ haplotype accounted for more than 0.700 in both AS patients and healthy controls. Notably, the rs1799964^T^-rs1800630^C^-rs1799724^C^-rs1800629^G^-rs361525^G^ haplotype was significantly associated with a reduced risk of AS (OR = 0.401, 95% CI: 0.268–0.601, *p* < 0.0001); the rs1799964^T^-rs1800630^C^-rs1799724^T^-rs1800629^G^-rs361525^G^ haplotype was significantly associated with an increased risk of AS (OR = 4.770, 95% CI: 2.956–7.697, *p* < 0.0001). Additionally, rs1799964^C^-rs1800630^C^-rs1799724^C^-rs1800629^G^-rs361525^A^ haplotype was less frequent in AS patients when compared with healthy controls (0.011 vs. 0.056, OR = 0.183, 95% CI: 0.040–0.829, *p* = 0.014).

### Predictive Classifier for AS

**Figure 2:**
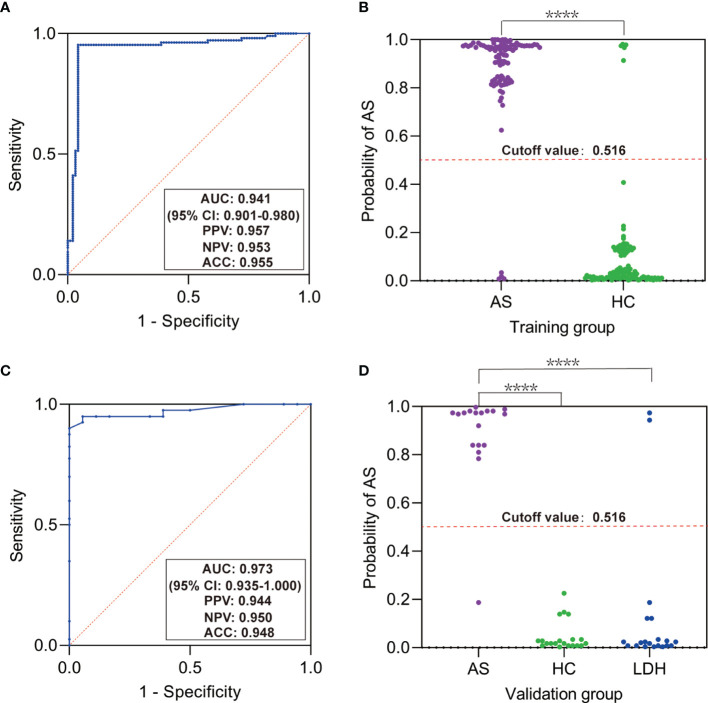
Predictive classifier for ankylosing spondylitis (AS) based on random forest algorithm. **(A)** Receiver-operating characteristic curve (ROC) for the training group. **(B)** Scatter plot of the probability of AS in the training group. **(C)** ROC for the validation group. **(D)** Scatter plot of the probability of AS in the validation group. AUC, area under ROC; PPV, positive predictive value; NPV, negative predictive value; ACC, accuracy; LDH, patients with lumbar disc herniation. ****p < 0.0001 by Mann–Whitney test or Kruskal–Wallis test.

**Figure 3:**
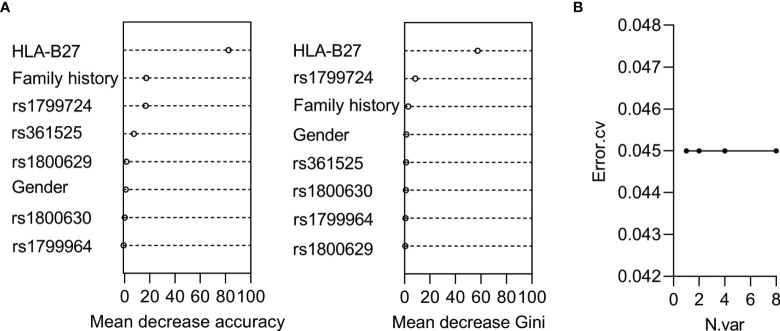
Analysis of the contribution of the variables for AS based on random forest algorithm. **(A)** The ranking chart of the two accuracy indicators. **(B)** The line chart of the relationship between numbers of variables and errors.

To investigate whether the discovered SNPs could be good complement for HLA-B27 in AS prediction, we used the five SNPs, HLA-B27, and other constant factors in life (gender and family history) to construct a classifier for AS based on random forest algorithm. The AUC was over 0.9 in both the training group (**Figure 2A**, AUC = 0.941, 95% CI: 0.901–0.980) and the validation group (**Figure 2C**, AUC = 0.973, 95% CI: 0.935–1.000). The cutoff value was 0.516 according to the AUC of the training group. Referring to the cutoff value, the AS patients could be specially distinguished from the healthy subjects and the patients with LDH (**Figures 2B, D**, *p* < 0.0001). However, these predicting results of the classifier were in complete consistency with classification according to HLA-B27 status (the results were not shown). To evaluate the contribution of each variable for the classification of AS, we used two comparative methods based on random forest algorithm. The two variable importance measures (mean decrease accuracy and mean decrease Gini) suggested that HLA-B27 ranked the first, while the two variables of family history and rs1799724 ranked second or third far from HLA-B27 (**Figure 3A**). The plot of variable selection and model prediction error further indicated that HLA-B27 could be the only variable for the predictive classifier (**Figure 3B**); thus, the five SNPs in *TNFα* including rs1799724 and rs361525 could not improve the prediction accuracy of HLA-B27.

### The Associations Between SNPs in *TNFα* and HLA-B27

Next, we wanted to know whether SNPs in *TNFα* were associated with HLA-B27. The subjects were divided into different groups for chi-square analysis, and no significant difference of the SNPs could be observed between AS patients and healthy controls in groups of HLA-B27 positivity or negativity. However, the genotype distribution of rs1799724 revealed a significant difference (*p* < 0.01) between HLA-B27 positivity and HLA-B27 negativity in each group (**Table 3**), and a strong association was found in the T allele of rs1799724 with HLA-B27 positivity in the group consisting of AS patients and healthy controls (T vs. C, OR = 6.431, 95% CI: 3.899–10.607, *p* < 0.0001, **Supplementary Table S5**). In addition, rs361525 showed a significant difference (*p* = 0.001) of genotype distribution between the individuals with HLA-B27 positivity and the individuals with HLA-B27 negativity when all the subjects were included (**Table 3**). As shown in **Supplementary Table S5**, the minor allele A of rs361525 demonstrated a strong association with HLA-B27 negativity (A vs. G, OR = 13.222, 95% CI: 1.722-101.542, *p* = 0.001).

**Table 3:** Genotype distributions of rs1799724 and rs361525 in different groups based on HLA-B27.

Group	Subgroup	rs1799724 (C > T)				rs361525 (G > A)		
		CC	CT	TT	*p*-value	GG	GA	*p*-value
HLA-B27 positivity	AS	21	53	15	0.670^a^	88	1	1^a^
	HC	0	4	1		5	0	
HLA-B27 negativity	AS	4	0	0	0.570^a^	3	1	0.438^a^
	HC	76	26	0		89	13	
AS	HLA-B27 positivity	21	53	15	**0.009** ^a^	88	1	0.085^a^
	HLA-B27 negativity	4	0	0		3	1	
HC	HLA-B27 positivity	0	4	1	**<0.0001** ^a^	5	0	1^a^
	HLA-B27 negativity	76	26	0		89	13	
AS & HC	HLA-B27 positivity	21	57	16	**<0.0001** ^b^	93	1	**0.001** ^b^
	HLA-B27 negativity	80	26	0		92	14	

a Fisher’s exact test.

b Chi-square test. The bold value showed the significance level less than 0.05.

**Figure 4:**
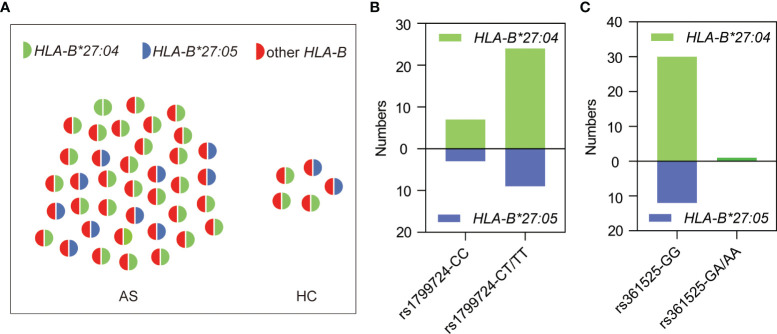
Genotyping results of 43 HLA-B27-positive subjects. **(A)** HLA-B genotyping results of AS patients (*n* = 38) and healthy controls (*n* = 5). **(B)** Genotype distribution of rs1799724 in the subjects carrying HLA-B*27:04 or HLA-B*27:05. **(C)** Genotype distribution of rs361525 in the subjects carrying HLA-B*27:04 or HLA-B*27:05. Other *HLA-B*, other alleles of *HLA-B* but none of HLA-B*27 alleles.

We randomly selected 38 HLA-B27-positive patients and all of the five positive healthy controls for sequencing analysis of *HLA-B* genotype. The results demonstrated that all the HLA-B27-positive subjects carried HLA-B*27:04 or HLA-B*27:05 (**Figure 4A**), and the genotype distributions of rs1799724 (**Figure 4B**) or rs361525 (**Figure 4C**) showed no statistical difference between HLA-B*27:04 and HLA-B*27:05 (*p* > 0.05). Nevertheless, higher proportion of genotypes with the T allele of rs1799724 and the G allele of rs361525 could be observed in the subjects carrying HLA-B*27:04 and HLA-B*27:05.

## Discussion

In addition to HLA-B27, finding more specific genetic markers is helpful to understand the pathogenesis of AS and predict the risk of AS at early time. In this study, we investigated the association between five SNPs in *TNFα* and the risk of AS in the Chinese Han population. No significant correlation was found between AS risk and rs1799964, rs1800630, and rs1800629. The T allele of rs1799724 was verified to significantly increase the risk of AS, while the A allele of rs361525 showed an association with the reduced AS risk. The rs1799964^T^-rs1800630^C^-rs1799724^T^-rs1800629^G^-rs361525^G^ haplotype revealed an association with higher risk of AS compared to others. Moreover, the two SNPs of rs1799724 and rs361525 were not independent factors of AS, which were suggested to be associated with HLA-B27. Due to the drawbacks especially the relatively small sample size, further validation with large sample size is needed.

Although the pathogenesis of AS is still not completely elucidated, many potential genetic risk factors for AS development were reported. Matrix metalloproteinase 3 (MMP-3) plays an important role in the degradation of extracellular matrix, which could promote the progress of chronic arthritis including AS (27). CT/TT genotype of rs522616 in *MMP*-3 and *MMP*-3-1171 6A/6A have been reported to be associated with the increased risk of AS (27, 28). Lines of evidence showed that AS patients experienced more activated T cells, and cytotoxic T-lymphocyte antigen-4 (CTLA-4) could negatively regulate T-cell activation (29). Nonetheless, the relationship between G allele of rs231775 in CTLA-4 and AS is controversial and more studies are needed (30). Th17 cells are involved in the pathogenesis of several autoimmune diseases including AS. C-C motif chemokine receptor 6 (CCR6) is a marker for Th17 cells; however, the SNPs in *CCR6* with AS susceptibility have not been validated (31, 32). In addition to the above potential pathogenesis, inflammatory cytokines and the related pathways are vital in the progress of AS. TNFα-axis is one of the key inflammatory pathways in AS, which has been evidenced by the good efficacy of TNF*α* blockade (33). Several studies have reported the association between SNPs in *TNFα* and AS susceptibility since some polymorphisms could alter the transcription and expression of TNFα (34), which could influence the fundamental role in immune responses. However, the associations of *TNFα* polymorphisms with AS risk remain controversial due to differences in ethnic populations, sample size, etc. (35). For SNPs interested in this study, rs1799964, rs1800630, and rs1800629 showed no statistical significance between SNPs and the risk of AS, which was in agreement with some reported case–control studies in Asian population (34, 36). However, another study in a Chinese Han population including 855 AS patients and 849 controls showed that the C allele of rs1799964 (OR = 0.60, 95% CI: 0.50–0.71, *p* < 0.0001), the A allele of rs1800630 (OR = 0.59, 95% CI: 0.48–0.72, *p* < 0.0001), and the A allele of rs1800629 (OR = 0.54, 95% CI: 0.39–0.74, *p* < 0.0001) were all linked to a reduced risk of AS (37). On the one hand, the possibility for the conflicting findings may be due to differences in sample size, and on the other hand, the study with a larger sample was needed to verify the repeatability of the correlation.

In 2005, it was first reported that the minor allele T of rs1799724 demonstrated a strong association with AS in the Chinese Han population (38). Then, whether rs1799724 is a susceptibility locus was controversial in different studies (34, 39). Our results further confirmed that individuals with the T allele could increase the risk of AS (*p* < 0.0001, the corrected *p* < 0.0001). Although rs361525 showed a null association with AS in most research (40), a study in Denmark indicated that individuals with GA genotype or AA genotype had a lower AS risk when compared to the GG genotype (*p* = 0.0024) (41). In the present study, we analyzed the genotype distribution and allele frequency of rs361525 and found that the A allele could play a protective role in a Chinese Han population. After haplotype analysis, the higher risk-associated haplotype (rs1799964^T^-rs1800630^C^-rs1799724^T^-rs1800629^G^-rs361525^G^) and the lower risk-associated haplotype (rs1799964^C^-rs1800630^C^-rs1799724^C^-rs1800629^G^-rs361525^A^) further demonstrated the strong association between the two SNPs (rs1799724 and rs361525) and AS.

Both rs1799724 and rs361525 locate in the promoter of *TNFα* and the biological effects have been validated in some studies. Compared with allele C of rs1799724, the transcriptional activity was higher in *TNFα* with allele T. Consequently, the levels of mRNA and protein of TNFα were significantly higher in the CT genotype as compared with the CC genotype (42). On the contrary, TNFα promoter with allele A of rs361525 showed a significantly decreased transcriptional activity when compared with the major allele T of rs361525 (43). Therefore, the association between the AS risk and the SNPs could be partly explained by the increased or decreased transcriptional activity of TNFα due to the specific alleles.

To screen out the susceptible individuals in early time, we explored to construct the predictive classifier for AS based on the genetic factors studied here and other constant features (such as gender) by logistic regression or random forest. Both methods for constructing the classifier showed that the optimal set of variables to predict AS risk was only HLA-B27, suggesting that rs1799724 and rs361525 contributed to AS not independently.

The above observations prompted us to ask if there were associations between HLA-B27 and the two SNPs of rs1799724 and rs361525. To our knowledge, few studies have discussed the relationship between the susceptibility loci and HLA-B27. Zhu et al. validated that the T allele of rs1799724 was associated with susceptibility to AS independently of *HLA-B27*; nevertheless, the genotype distribution of rs1799724 deviated from HWE in the control group (44). A study in Brazilian population with HLA-B27 negativity found no association between two SNPs (rs1800629 and rs361525) in *TNFα* and AS (45). In the present study, all the interested SNPs obeyed HWE and no association between the SNPs and the risk of AS was observed in HLA-B27 positive or negative individuals. However, the T allele of rs1799724 and the G allele of rs361525 correlated strongly with HLA-B27 positivity in the group including all the AS patients and healthy controls (both *p* < 0.01). In terms of gene location, *TNFα* locates on the same chromosome as HLA-B*27 (46). Further sequencing results showed that all the tested HLA-B27-positive samples carried HLA-B*27:04 or HLA-B*27:05, suggesting a potential LD between *TNFα* and HLA-B*27.

In conclusion, the T allele of rs1799724 was associated with an increased risk of AS, while the A allele of rs361525 contributed to the decreased risk of AS in the Chinese Han population. However, the association between the two SNPs and AS risk might be attributed to HLA-B*27:04 and HLA-B*27:05. Although the present study has an obvious limitation of small sample size, it indicates the importance of taking HLA-B27 and HLA-B*27 into consideration in AS association studies. HLA-B27 is still an irreplaceable genetic marker in the prediction of AS, finding that the susceptibility loci contributing to AS independently will be of great significance in the future.

## Data Availability Statement

The original contributions presented in the study are included in the article/**Supplementary Material**. Further inquiries can be directed to the corresponding authors.

## Ethics Statement

The studies involving human participants were reviewed and approved by the Ethics Committee of First People’s Hospital of Nantong City. Written informed consent to participate in this study was provided by the participants’ legal guardian/next of kin.

## Author Contributions

Conceptualization, methodology, and original draft preparation: LS, NS, and YG. Critical analysis of the manuscript: HL and LG. Acquisition of data: WW, BZ, and QH. Funding acquisition: XW, NS, YG, and WW. Project administration: LS and XW. All authors contributed to the article and approved the submitted version.

## Funding

This research was supported by Nantong Science and Technology Bureau (No. JC2020037), the Health Commission of Nantong City (No. MB2021015, No. QA2021013, and No. QA2021014), and Jiangsu Research Hospital Association for Precision Medication (No. JY202110).

## Conflict of Interest

The authors declare that the research was conducted in the absence of any commercial or financial relationships that could be construed as a potential conflict of interest.

## Publisher’s Note

All claims expressed in this article are solely those of the authors and do not necessarily represent those of their affiliated organizations, or those of the publisher, the editors and the reviewers. Any product that may be evaluated in this article, or claim that may be made by its manufacturer, is not guaranteed or endorsed by the publisher.
