# Supplementary material for: The Associations of rs1799724 and rs361525 With the Risk of Ankylosing Spondylitis Are Dependent on HLA-B27 Status in a Chinese Han Population
Source: Front Immunol. 2022 Apr 5;13:852326. doi: 10.3389/fimmu.2022.852326 (PMC9016113; doi:10.3389/fimmu.2022.852326)
Supplement: Supplementary file 1 [file Table_1.docx]

Supplementary Material

# Supplementary Tables

**Table S1** Sequences of primers and probes for five SNPs in *TNFα*.

| **Location** | **Name** | **Sequence (5'-3')** |
| --- | --- | --- |
| rs1799964 | FP | CGCCAGACTGCTGCAGGG |
|  | RP | CCCCCGCCCCTCCAG |
|  | UP | GAAGCAAAGGAGAAGCTGAGAAGAA |
|  | DP-T | CGCGCCGAGG**T**GAAGGAAAAGTCAGGG-PO_3_ |
|  | DP-C | ACGGACGCGGAG**C**GAAGGAAAAGTCAGGG-PO_3_ |
| rs1800630 | FP | GGTAGGAGAATGTCCAGGGCTATGG |
|  | RP | GGAGGCTCTTTCACTCCCTGGG |
|  | UP | TCGAGTATGGGGACCCCCT |
|  | DP-C | CGCGCCGAGG**C**CTTAAYGAAGACAGGG-PO_3_ |
|  | DP-A | ACGGACGCGGAG**A**CTTAAYGAAGACAGGG-PO_3_ |
| rs1799724 | FP | GTCCAGGGCTATGGAAGTCGAGT |
|  | RP | GGAGGCTCTTTCACTCCCTGGG |
|  | UP | ATGGGGACCCCCCCTTAAA |
|  | DP-C | CGCGCCGAGG**C**GAAGACAGGGCCA-PO_3_ |
|  | DP-T | ACGGACGCGGAG**T**GAAGACAGGGCCA-PO_3_ |
| rs1800629 | FP | TGGTCCCCAAAAGAAATGGAGGCA |
|  | RP | GGTCTTCTGGGCCACTGACTGA |
|  | UP | GAGGCTGAACCCCGTCCA |
|  | DP-G | CGCGCCGAGG**C**CATGCCCCTCAAAA-PO_3_ |
|  | DP-A | ACGGACGCGGAG**T**CATGCCCCTCAAAAC-PO_3_ |
| rs361525 | FP | ACAAATCAGTCAGTGGCCCAGAAGA |
|  | RP | ACAAGCATCAAGGATACCCCTCACA |
|  | UP | TCCCCATCCTCCCTGCTCA |
|  | DP-G | CGCGCCGAGG**C**GATTCCGAGGGGG-PO_3_ |
|  | DP-A | ACGGACGCGGAG**T**GATTCCGAGGGGG-PO_3_ |
|  | FRET-1 | FAM-TCTT (BHQ1)AGCCGGTTTTCCGGCTAAGACTCCGCGTCCGT-C6-NH_2_ |
|  | FRET-2 | VIC-TCTT (BHQ1)AGCCGGTTTTCCGGCTAAGACCTCGGCGCG-C6-NH_2_ |

FP, forward primer; RP, reverse primer; UP, upstream probe; DP, downstream probe for specific allele; FRET, fluorescence resonance energy transfer. The red colored text showed the alleles of each SNP.

**Table S2** Demographic and clinical characteristics of ankylosing spondylitis (AS) patients with and without family history.

| **Characteristic** | **With family history (n = 14)** | **Without family history (n = 79)** | ***p*-value** |
| --- | --- | --- | --- |
| Gender (male, %) | 78.5 | 68.4 | 0.651 |
| Age at enrollment (years, median (IQR)) | 31.00 (13.00) | 40.00 (19.00) | 0.150 |
| Age at symptom onset (years, median (IQR)) | 25.00 (7.50) | 33.00 (19.00) | **0.005** |
| Age at diagnosis (years, median (IQR)) | 28.00 (11.75) | 38.00 (19.00) | **0.024** |
| BMI (kg/m^2^, mean ± SD) | 24.6 ± 3.7 | 23.7 ± 3.2 | 0.359 |
| HLA-B27 positivity (%) | 100 | 94.9 | 1 |
| ESR (mm/h, median (IQR)) | 10.00 (12.50) | 20.00 (45.00) | 0.097 |
| Hs-CRP (mg/L, median (IQR)) | 7.32 (23.73) | 13.65 (44.52) | 0.364 |
| BASDAI (score, median (IQR)) | 3.60 (2.00) | 3.00 (2.00) | 0.550 |
| BASFI (score, median (IQR)) | 2.90 (2.78) | 2.80 (1.90) | 0.779 |

BMI, body mass index; ESR, erythrocyte sedimentation rate; Hs-CRP, high-sensitivity C-reactive protein; BASDAI, bath ankylosing spondylitis disease activity index; BASFI, bath ankylosing spondylitis functional index. The bold value showed the significance level less than 0.05.

**Table S3** *P* values of Hardy-Weinberg equilibrium test for patients and healthy controls**.**

| **SNP** | **Variation** | ***p*-value** | |
| --- | --- | --- | --- |
|  |  | **AS (n = 93)** | **HC (n = 107)** |
| rs1799964 | T > C | 0.060 | 0.503 |
| rs1800630 | C > A | 0.580 | 0.393 |
| rs1799724 | C > T | 0.140 | 0.290 |
| rs1800629 | G > A | 0.706 | 0.503 |
| rs361525 | G > A | 0.917 | 0.504 |

AS, AS patient; HC, healthy controls. The bold value showed the significance level less than 0.05.

**Table S4** Genetic models of rs1799724 in AS patients and healthy controls.

| **Model** | **Genotype** | **AS n (%)** | **HC n (%)** | ***p*-value^a^** | **OR (95% CI)** |
| --- | --- | --- | --- | --- | --- |
| Over-dominant | CC+TT | 40 (43.0) | 77 (72.0) |  | Reference |
|  | CT | 53 (57.0) | 30 (28.0) | **< 0.0001** | 3.401 (1.888-6.127) |
| Dominant | CC | 25 (26.9) | 76 (71.0) |  | Reference |
|  | CT+TT | 68 (73.1) | 31 (29.0) | **< 0.0001** | 6.668 (3.587-12.398) |
| Recessive | TT | 15 (16.1) | 1 (0.9) | **< 0.0001** | 20.385 (2.637-157.595) |
|  | CC+CT | 78 (83.9) | 106 (99.1) |  | Reference |
| Co-dominant | CC | 25 (26.9) | 76 (71.0) |  | Reference |
|  | CT | 53 (57.0) | 30 (28.1) | **< 0.0001** | 5.371 (2.843-10.147) |
|  | TT | 15 (16.1) | 1 (0.9) | **< 0.0001** | 45.600 (5.731-362.845) |

^a^Chi-square test. OR, odds ratio; CI, confidence interval. The bold value showed the significance level less than 0.05.

**Table S5** Allele frequencies of rs1799724 and rs361525 in different groups based on HLA-B27.

| **Group** | **Subgroup** |  | **rs1799724 (C > T)** | | | | | | **rs361525 (G > A)** | | |
| --- | --- | --- | --- | --- | --- | --- | --- | --- | --- | --- | --- |
|  |  |  |  | **C** | **T** | ***p*-value** |  | **G** | | **A** | ***p*-value** |
| HLA-B27 positivity | AS |  |  | 95 | 83 | 0.618^a^ |  | 177 | | 1 | 1^b^ |
|  | HC |  |  | 4 | 6 |  |  | 10 | | 0 |  |
| HLA-B27 negativity | AS |  |  | 8 | 0 | 0.600^b^ |  | 7 | | 1 | 0.427^b^ |
|  | HC |  |  | 178 | 26 |  |  | 191 | | 13 |  |
| AS | HLA-B27 positivity |  |  | 95 | 83 | **0.026**^a^ |  | 177 | | 1 | 0.084^b^ |
|  | HLA-B27 negativity |  |  | 8 | 0 |  |  | 7 | | 1 |  |
| HC | HLA-B27 positivity |  |  | 4 | 6 | **< 0.0001**^a^ |  | 10 | | 0 | 1^b^ |
|  | HLA-B27 negativity |  |  | 178 | 26 |  |  | 191 | | 13 |  |
| AS & HC | HLA-B27 positivity |  |  | 99 | 89 | **< 0.0001**^a^ |  | 187 | | 1 | **0.001**^a^ |
|  | HLA-B27 negativity |  |  | 186 | 26 |  |  | 198 | | 14 |  |

^a^Chi-square test; ^b^Fisher's exact test. The bold value showed the significance level less than 0.05.
